# Neuroinflammation‐Associated Optic Structural–Functional Degeneration in Early Diabetic Optic Neuropathy

**DOI:** 10.1111/1753-0407.70198

**Published:** 2026-03-21

**Authors:** Jingyuan Zhu, Jinyuan Wang, Haihan Zhang, Mingyang Wang

**Affiliations:** ^1^ Beijing Tongren Eye Center, Beijing Institute of Ophthalmology, Beijing Tongren Hospital Capital Medical University Beijing China; ^2^ School of Clinical Medicine Tsinghua Medicine, Tsinghua University Beijing China

**Keywords:** diabetic optic neuropathy (DON), ocular structure‐functional imaging, peripapillary retinal thickness, retinal neurodegeneration, visual behavior

## Abstract

**Background:**

Diabetic optic neuropathy (DON) is characterized by optic nerve degenerative changes, which may progress to optic atrophy and even blindness. Although DON is relatively common, its early diagnosis remains challenging. This study employed a noninvasive in vivo imaging combined histopathological assessment system to investigate changes in optic structural–functional degeneration in DON models.

**Methods:**

Animals were divided into experimental group (8 db/db mice) and control group (8 db/m mice). Blood glucose, body weight, intraocular pressure, peripapillary retinal nerve fiber layer (RNFL), total retinal thickness (TRT), and visual behavior were at 2, 3, 4, 5 and 8 months old. Furthermore, histopathological and molecular validations were performed on the peripapillary retinal tissue.

**Results:**

Across the 6‐month follow‐ups, db/db mice remained hyperglycemic and obese. Compared with the control group, db/db mice showed significant thinning of the peripapillary RNFL and TRT, and pyroptosis of retinal gangolion cells at 3 months old, suggesting optic nerve degeneration. Meanwhile, decreased visual acuity and increased contrast sensitivity thresholds confirmed the establishment of an early DON model. Thinning of the TRT at 8 months old was found to be linearly correlated with decreased visual acuity, but not with contrast thresholds in the DON group. No significant differences of intraocular pressure were found between groups at any time point.

**Conclusions:**

Db/db mice provide a reliable model for early DON study. Retinal structural impairment was closely associated with visual dysfunction, underscoring the link between neurodegeneration and functional loss. These findings may facilitate early diagnosis and treatment of DON.

## Highlights

A noninvasive in vivo structural‐functional assessment system that incorporates optical coherence tomography and visual behavior detection, combined with histopathology validation, was applied to successfully establish an early diabetic optic neuropathy (DON) model. Db/db mice exhibited early thinning of the peripapillary retinal nerve fiber layer and total retinal thickness, along with retinal ganglion cell pyroptosis as 3 months old. These changes were accompanied by decreased visual acuity and increased contrast sensitivity thresholds. At 8 months old, of the retinal thinning linearly correlated with decreased visual acuity, highlighting the close link between retinal structural degeneration and visual dysfunction. This model provides a reliable platform for exploring DON pathogenesis and supports early diagnosis and intervention strategies.

## Graphical abstract

OCT imaging assessments and visual tests, which were validated by pyroptosis of retinal ganglion cells (RGC) and upregulation of NLRP3 and GSDMD expression, established an early structure‐function‐pathology diabetic optic neuropathy (DON) model in high glucose & obesity mice. Early retinal nerve fiber layer (RNFL)/total retinal thickness (TRT) thinning were associated with visual dysfunction, providing new ideas for early diagnosis and intervention.

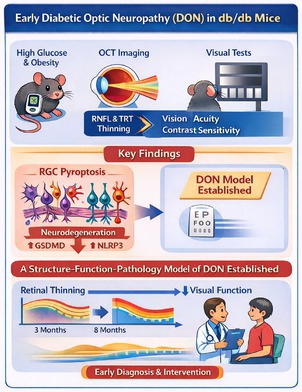

## 1 Introduction

Optic nerve degeneration is caused by demyelination of retinal ganglion cell (RGC) axons or the retrobulbar optic nerve. It is a progressive chronic process that ultimately culminates in optic atrophy and loss of visual function. The Axons of RGCs constitute the optic nerve, which is part of the central nervous system. Since central neurons generally lack regenerative capacity, degenerative changes in RGCs often lead to irreversible damage and eventually blindness [1].

Among the multiple forms of RGC death, pyroptosis has recently drawn attention as a critical mechanism involved in the pathogenesis of various neurodegenerative disorders. The canonical pathway is initiated by activation of inflammasomes, such as NLRP3, which subsequently cleave and activate pro‐caspase‐1 to generate active caspase‐1 [2]. Activated caspase‐1 then cleaves gasdermin D (GSDMD), resulting in membrane pore formation and eventual cell rupture [3]. Given these mechanisms, it remains unclear whether pyroptosis contributes to RGC injury in DON.

Optic nerve degeneration can be classified as primary—usually associated with glaucoma or hereditary optic neuropathies, or secondary—commonly tied to systemic diseases, age‐related eye diseases, brain inflammation, trauma, tumors, etc. In secondary forms, diabetic optic neuropathy (DON) has a high incidence of optic nerve and retinal degenerative changes observed in 4.7% of patients with 1–5 years of diabetes and 7.9% in patients with 6–10 years of diabetes [4]. However, early diagnosis remains difficult due to the unclear pathogenesis of DON.

While DON and diabetic retinopathy (DR) are both commonly encountered in clinical practice, studies have found that their occurrence is not parallel. Patients with pre‐proliferative DR exhibited a higher incidence of papilledema and anterior ischemic optic neuropathy than patients with proliferative DR [5]. Moreover, diabetic patients with or without DR displayed abnormal visual evoked potentials, and those without retinopathy presented significantly higher contrast sensitivity than those with retinal involvements [6]. These findings suggested that diabetic patients have abnormalities in vision‐related neural pathways other than DR. In addition, some diabetic patients had visual field defects without detectable retinopathy, indicating that DON may precede microvascular changes and clinical manifestations caused by DR [7]. This makes DON one of the major blinding eye diseases. Thus, our study focused on DON occurring prior to DR.

Although we have previously conducted in‐depth studies on DR, relatively few animal experiments and controlled studies have addressed ocular structure and function of early optic neurodegeneration in DON. Therefore, effective early interventions of DON remain limited. Several clinical studies have shown that DON impairs visual function—visual acuity, visual fields, and visual evoked potentials [8]. Cross‐sectional studies of structural changes across retinal thickness have reported inconsistent findings at comparable ages and follow‐up times. Early studies of db/db mice monitored from 8 weeks of age, whose experimental and control groups were identical to our study, showed no significant differences in total retinal thickness (TRT) between groups at 11 weeks [9]. Whereas other studies identified significant TRT reductions of db/db mice at 8 weeks compared with controls [10]. To address these inconsistencies, we performed longitudinal TRT measurements in a db/db mouse model of early DON.

By employing a noninvasive in vivo assessment system integrating optical coherence tomography, visual behavioral detection and tonometry, combined with histopathology validation, this study provided a well‐designed, model‐controlled structure‐function analysis at specific follow‐up points. Such an approach is expected to advance early manifestations and progression of DON, with important implications for timely diagnosis and intervention.

## 2 Methods

### 2.1 Animal Models and Groups

The animal experimental protocol and all experimental procedures were approved by the Animal Care and Use Committees and Ethics Committees at Capital Medical University. Transgenic mice on the C57BLKS/J background were used in the experimental group, including eight 2‐month‐old male db/db mouse models (BKS.Cg‐Dock7m +/+ Leprdb/J, Dock7m wild‐type, leptin receptor knockout, Leprdb‐ homozygous). These offspring are leptin receptor‐deficient models that spontaneously develop diabetes and obese. The control group included eight age‐ and sex‐matched db/m mouse models from the same colony as the experimental group (BKS.Cg‐Dock7m +/+ Leprdb/J, Dock7m heterozygous, Leprdb heterozygous). These were non‐leptin receptor knockout model, which were used to breed db/db offsprings as normal phenotypes (https://www.jax.org/news‐and‐insights/jax‐blog/2012/march/if‐im‐not‐obese‐im‐misty). Mice were maintained under a 12‐h light/12‐h dark cycle (6:30 AM to 6:30 PM simulated daytime) at 21°C–24°C in standardized incubators (≤ 5 mice/cage) with food and water provided ad libitum. Husbandry was conducted uniformly by trained staff.

Retinal structure was assessed by optical coherence tomography (OCT), visual function was evaluated by optokinetics, intraocular pressure (IOP) was measured using rebound tonometry at baseline (at 2 months old) and follow‐ups (at 3, 4, 5, and 8 months old). Besides, cell pyroptosis and inflammation‐related proteins were detected.

### 2.2 Physiological Parameter Measurements

All mice in the two groups were monitored for rapid blood glucose and body weight at all time points. Random blood glucose was obtained via tail vein acupuncture using a rapid glucose meter. Sampling was completed promptly to minimize physiological fluctuations caused by anesthesia. The weight of freely moving mice was individually measured using an electronic scale.

IOP was part of the physiological measurements in this study. Binocular IOP was measured by a contact rebound tonometer (TonoLab, Icare, Finland). For each eye, six single readings were taken, the highest and lowest discarded, and the mean of the remaining four values was recorded. To keep the IOP as close as possible to the physiological state, IOP measurements were conducted brief inhalation anesthesia.

### 2.3 Peripapillary Retinal Imaging and Measurements

Binocular scans were performed using spectral domain OCT (Envisu R2210, Leica Microsystems, Germany) to obtain retinal images centered on the optic disc. The segmented retinal images were performed using customized software based on validated algorithms [11] that previously applied in related studies [12]. A ring sampling belt between two concentric circles with radius of 0.234 to 0.324 mm centered on the mouse optic nerve head (ONH) was selected as the range of interest (ROI) to obtain peripapillary retinal thickness. The distance between the inner limit membrane (ILM) and Bruch membrane (BM) was selected to determine TRT. Retinal nerve fiber layer (RNFL) thickness was separately quantified.

### 2.4 Assessments of Visual Behavior

Visual function was assessed using OptoDrum virtual‐reality optokinetic system (Striatech, Germany). Visual acuity was represented by spatial frequency, and contrast sensitivity was represented by contrast thresholds (reciprocal). Animals were tested awake. The visual function, behavioral pattern and psychophysical perception of animals were systematically integrated. Stimulus patterns of moving gratings with varying frequencies and gray levels were presented to elicit reflexive head and eye movements.

Through editing different computer stimulus patterns, the quantitative data of motion was transmitted to the supporting computer recording platform and analysis system [13]. Each mouse was acclimated for 5 min, and then followed the gratings in a randomly system‐generated sequence, which rotated clockwise or counterclockwise directions to test left or right eyes, respectively. Testing ended when consistent head tracking ceased. The results showed that lower spatial frequency indicated poor visual acuity, and higher contrast threshold indicated worse visual function.

### 2.5 Histopathological Examinations

At 2 and 3 months of age, db/db mice and db/m mice (not included in expremental groups) were euthanized, and retinal tissues surrounding the ONH were harvested for sectioning. RGCs were immunolabeled using Brn3a antibody and examined under a fluorescence microscope (DMi8, Leica, Germany) to observe numbers, activity, and morphological changes of RGC. Given that diabetes could induce inflammatory injury to the optic nerve and retina, particular evaluation was given to whether RGCs exhibited typical features of pyroptosis (inflammatory cell death) in the experimental group. Next, Western Blot analysis was performed to evaluate the expression levels of the apoptotic protein GSDMD and inflammation‐related proteins NLRP3 in the retinal tissue, to investigate diabetes‐induced inflammatory response and RGC pyroptosis.

### 2.6 Statistical Analysis

Descriptive statistic analysis and *t*‐test were used to compare the blood glucose and body weight between the two groups. Peripapillary retinal thickness, visual acuity, contrast threshold, and IOP were analyzed by three‐way ANOVA (GraphPad Prism 9.1.0, La Jolla, USA), considering three factors of the two groups, right and left eyes and time points, followed by Tukey's post hoc test. Correlations between retinal thickness and visual acuity or contrast sensitivity in the experimental group were performed using Pearson correlation analysis. In this study, data are presented as mean ± standard error (SE), and statistical significance was defined as *p* ≤ 0.05.

## 3 Results

### 3.1 Changes in Blood Glucose, Body Weight and IOP

**FIGURE 1:**
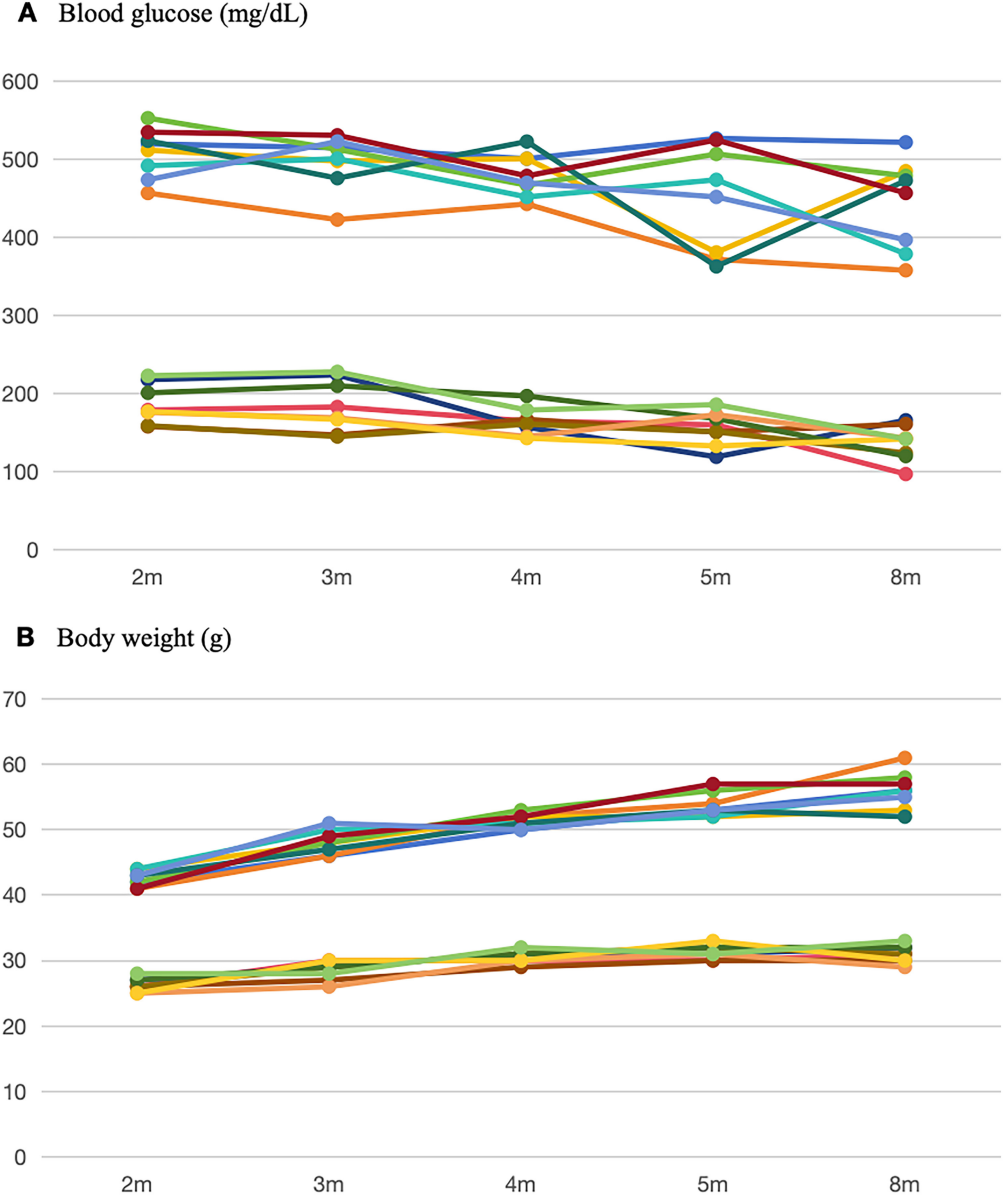
Blood glucose (A) and body weight (B) over time. The top cluster represents the blood glucose (A) or body weight (B) in the db/db mice, respectively; the lower cluster represents the blood glucose (A) or body weight (B) in the db/m mice, respectively.

Random blood glucose measurements were between 365–562 mg/dL in db/db mice (experimental group) and 98–228 mg/dL in db/m mice (control group) during follow‐ups from 2 to 8 months of age. Figure 1A shows the curves of blood glucose over time for each mouse. The upper cluster of curves in the figure shows that the db/db mice consistently maintained hyperglycemia with blood glucose levels greater than 350 mg/dL, whereas the lower cluster of curves shows that the blood glucose of the control mice remained less than 350 mg/dL. The *t*‐test showed a significant inter‐group difference in blood glucose (*p* < 0.001).

The mean body weight in the experimental group increased from 42.50 ± 1.19 g at 2 months old to 56.00 ± 2.83 g at 8 months old, while the control group increased from 26.25 ± 1.04 g to 31.00 ± 1.31 g (Figure 1B). The individual body weight in both groups gradually increased over time. The top cluster in the figure, shows that the weight of each diabetic mouse was greater than that of the control group from the start of the experiment. The weight gain trend of the experimental group was greater than that of the control group. Differences between groups were significant (*p* < 0.0001). Furthermore, db/db mice exhibited a distinct abdominal obesity phenotype, whereas db/m mice were normal‐sized.

Comparisons of IOP between the two groups over time showed fluctuation between 9.8 and 20.8 mmHg in both groups throughout the study, and the fluctuation range of IOP for each mouse was between 0 and 8.9mmHg. When compared within the diabetic group, IOP increased at 8 months old (*p* < 0.05) compared with the baseline. However, no significant difference of IOP were observed between groups. Our results observed IOP variations only within the db/db group, and no significant differences within the db/m group at any time point. IOP remained within the physiological range in both groups, and no direct correlation between RNFL defect and IOP was identified.

### 3.2 Retinal Structure Changes Around ONH

**FIGURE 2:**
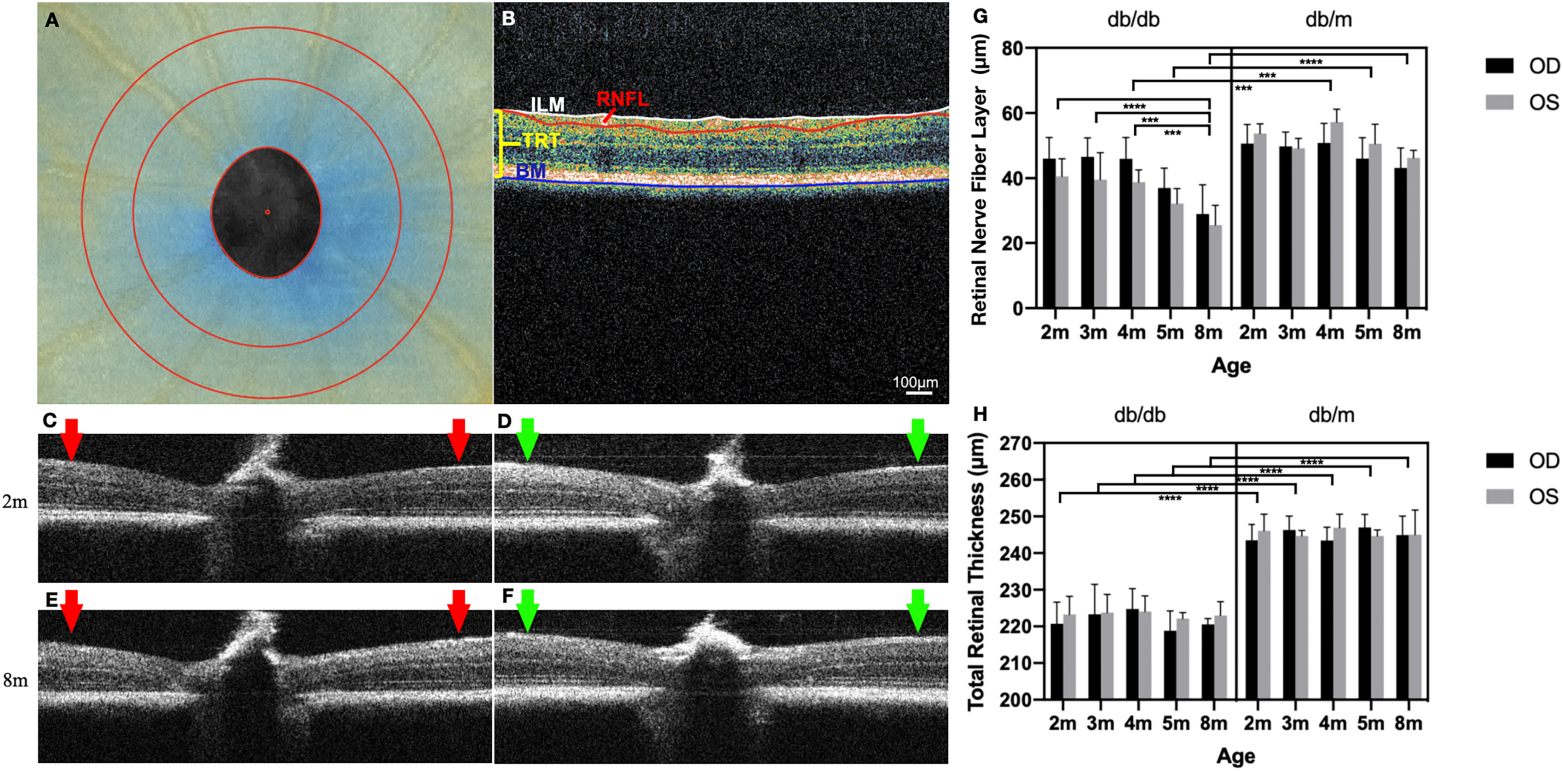
Peripapillary retinal imaging and thickness changes over time. A representative *en‐face* OCT image with ROI (A). A retinal cross‐sectional image with segmentations (B): The white line is the inner limiting membrane (ILM), the blue line is defined as the outer boundary of the retina (Bruch membrane, BM), the distance between the white line and the blue line represents the total retinal thickness (TRT), and the distance between the white line and the red line represents the retinal nerve fiber layer (RNFL) thickness. Comparisons of the morphology and thickness of representative B‐scan images in the diabetic group (C and E) and the nondiabetic group (D and F) at 2 months old (C and D) and 8 months old (E and F), respectively. Changes in RNFL thickness (G) and TRT (H) over time in both groups of mice. (In panels G and H, 2–8 m: 2 months old ~8 months old; OD: Right eye, OS: Left eye. Columns represent means and upper bars represent SE. ***p < 0.001, **** *p* < 0.0001).

The transparency of optical medium were continuously observed using microscope. We found no cornea, aqueous humor or lens opacities, nor secondary ocular complications of diabetes such as iris and retinal neovascularization. With clear optical media, retinal en‐face and cross‐sectional OCT images centered on ONH (Figure 2A,B) were obtained at all time points.

Compared the representative B‐scan images of diabetic and control groups, BM of both groups was in the same horizontal line, while limiting membrane (ILM) was different (Figure 2, red arrows in C&E, compared green arrows in D&F). The morphological changes illustrated marked retinal thinning in db/db mice compared with db/m mice.

Quantitatively, relative to the control group, RNFL thickness significantly decreased from 4 (*p* < 0.001) to 8 months of age (*p* < 0.0001, Figure 2G) in the diabetic group. Within diabetic group, RNFL progressive thinned from 43.30 ± 5.99 μm at 2 months to 27.18 ± 7.13 μm at 8 months of age, and was significant at 2, 3, and 4 months old compared with 8 months old (*p* < 0.0001, *p* < 0.001, *p* < 0.001, respectively). Moreover, TRT of diabetic mice was consistently thinner than that of controls across all time points (*p* < 0.0001 for all, Figure 2H). Intra‐group comparisons showed minimal changes that TRT was 221.98 ± 5.27 μm at baseline and 221.78 ± 2.94 μm at 8 months old in diabetic mice, while 244.80 ± 4.31 μm at baseline and 244.94 ± 5.60 μm at 8 months old in control mice (*p* > 0.05). No apparent difference in RNFL thickness or TRT was found between right eye (OD, black bar) and left eye (OS, gray bar) at each same time point in the same mouse. No statistically significant RNFL thickness or TRT change was observed within nondiabetic group.

### 3.3 Visual Function Changes

**FIGURE 3:**
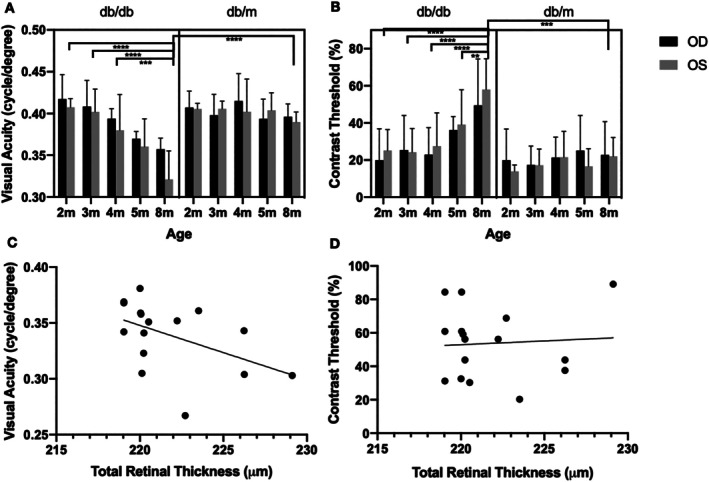
Visual acuity and contrast threshold and their correlation with peripapillary total retinal thickness. Changes in visual acuity (A) and contrast sensitivity threshold (B) over time in both groups of mice. Correlation between peripapillary total retinal thickness and visual acuity (C) and contrast threshold (D), respectively. (In panels A and B, 2–8 m: 2 months ~8 months old; OD: Right eye, OS: Left eye. The bar represents the mean, and the horizontal line above represents the SE. **p < 0.01, ***p < 0.001, ****p < 0.0001).

Under the condition of transparent optical media, this experiment showed that the visual acuity of the diabetic group decreased and the contrast sensitivity threshold increased during follow‐ups compared with baseline. But the visual functions of db/m mice remained relatively stable (Figure 3).

When compared between groups, the visual acuity of diabetic mice was significantly lower than that of control mice at 8 months old (Figure 3A, *p* < 0.01). When compared within the diabetic group, visual acuity gradually decreased from 0.412 ± 0.021 cycles/degree (c/d) at 2 months to 0.338 ± 0.030 c/d at 8 months old. There was no significant difference between the left and right eyes of the same mouse and at the same time point (*p* < 0.01 or *p* < 0.001). In contrast, the nondiabetic group showed no significant changes throughout the experiment, with visual acuity of 0.406 ± 0.014 c/d at 2 months and 0.392 ± 0.014 c/d at 8 months old (*p* > 0.05).

Contrast threshold in the db/db mice was significantly higher than that of db/m mice at 8 months old (Figure 3B, *p* < 0.05). When compared within the db/db mice, the contrast threshold increased from 22.53% ± 13.72% at 2 months to 53.71% ± 20.29% at 8 months old, and was significantly higher at 8 months old than at each follow‐up time point from 2 to 5 months old (*p* < 0.01 or *p* < 0.0001). However, the db/m mice showed no significant change with values of 17.32% ± 14.70% at 2 months and 21.13% ± 13.79% at 8 months (*p* > 0.05). There was no significant difference between the left eye and the right eye either.

Correlation analysis of bilateral eyes of all diabetic mice at the end point (8 months old) showed that increased TRT correlated positively with decreased visual acuity (Figure 3C, *p* ≤ 0.05), but not with contrast threshold (Figure 3D, *p* > 0.5). There was no linear correlation between RNFL thickness and visual acuity or contrast threshold.

### 3.4 Histopathological and Molecular Changes

**FIGURE 4:**
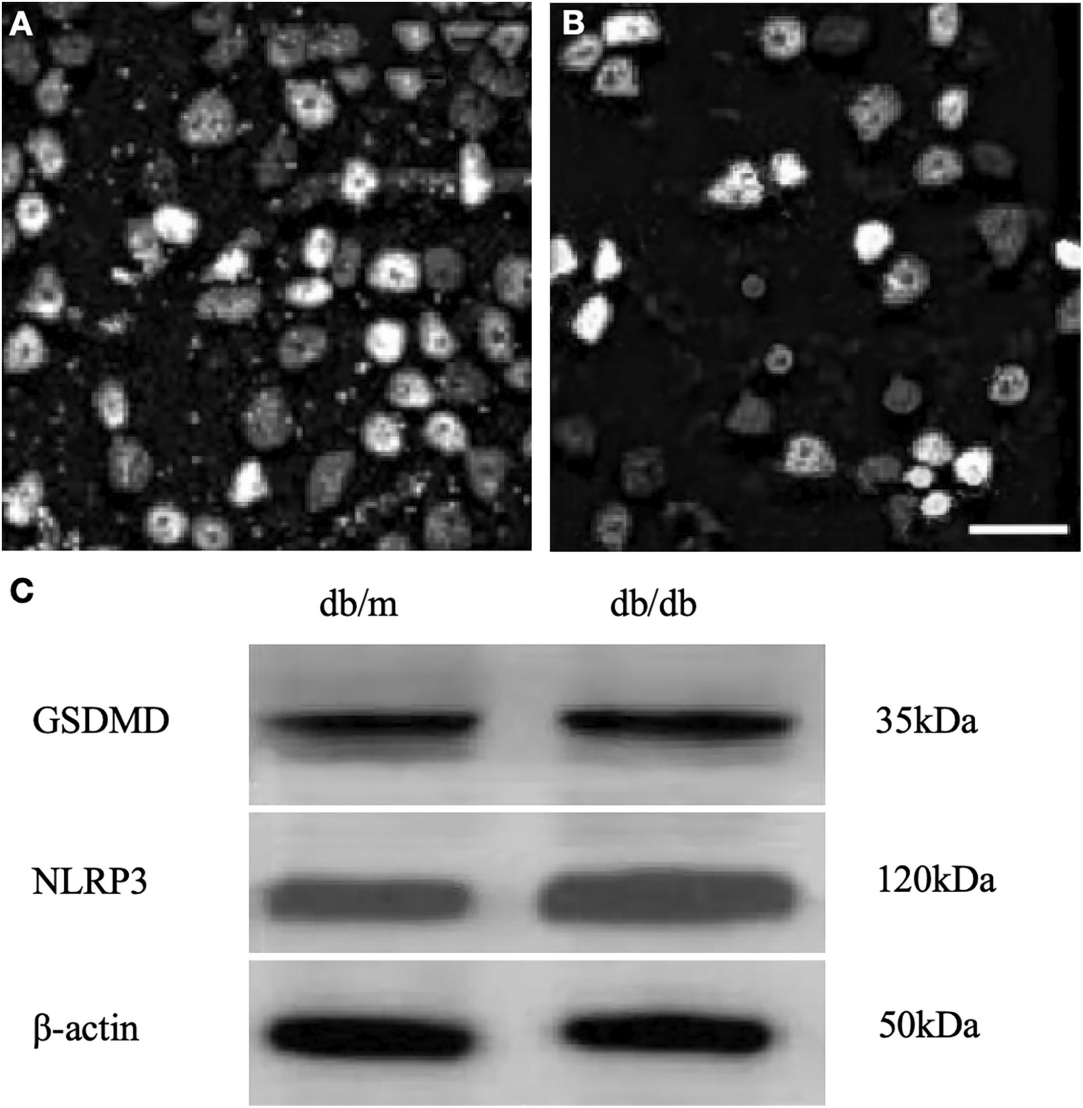
Retinal sections and western blot analysis. Representative images of RGCs in (A) db/m mice (non‐diabetic control group) and (B) db/db mice (diabetic group), (scale bar: 20 μm). (C) Western blot results showing thicker and more intense electrophoretic bands of GSDMD and NLRP3 proteins in db/db mice (unit: kDa), indicating increased expression levels; *β*‐actin served as internal control.

Three‐month‐old db/db mice exhibited a reduction in RGC counts compared with control mice (Figure 4A,B). Cells appeared swollen and enlarged, with membrane protrusions and partial cytoplasmic leakage, consistent with morphological changes of pyroptosis (Figure 4B), in which cells progressively swell until plasma membrane rupture occurs. Western blot analysis confirmed significantly elevated expression levels of GSDMD and NLRP3 inflammasome in db/db mice compared with db/m controls (Figure 4C). These findings indicated that pyroptosis and inflammatory injury of RGCs in diabetic mice contributed to early optic neurodegeneration, establishing a reliable early DON model.

## 4 Discussion

Diabetes mellitus is a chronic systemic disease which commonly causes optic neuropathy and irreversible visual impairments [5]. DON represents a specific form of optic neurodegeneration resulting from diabetes‐induced metabolic insults. Persistent hyperglycemia promotes oxidative stress and inflammation of the optic nerve. Diabetes induced injury can spread along the macula and through the lentigo bundle, developing into neural and retinal degenerative disease, leading to neural injury that can extend from the macular region along the lentigo bundle to the optic nerve. As the prevalence of Type II diabetes is rapidly increasing worldwide, DON has become one of the leading causes of vision loss. Retinal neurodegeneration occurs early in diabetes, even before clinically detectable DR. This study focused on diabetes‐induced optic neurodegenerative changes in retinal structure and visual function, as markers of early DON prior to the DR.

Most of the past researches evaluated the diabetic retinal neurodegeneration from the perspectives of tissue sections (morphology) and electrophysiology (visual function) [14], while our study identified the early stage of DON through non‐invasive OCT imaging (structure) and optic optokinetics (visual function), and validated by histopathology. Previous studies focused more on the microvascular changes of DR such as capillaries and its pericytes, without conducting in‐depth research on the neuropathy of diabetes [10].

### 4.1 Physiological Parameters and DON Model Establishment

Previous studies by Jackson Laboratory showed that mice with glucose concentrations greater than 350 mg/dL in peripheral venous whole blood can be used to define diabetes. Consistent with their further studies, our db/db mice present as a typically obese phenotype after leptin receptor knockout, while db/m controls maintained normal glucose levels and body weight. The C57BLKS/J background used in this study preserved a stable hyperglycemic phenotype throughout follow‐ups, avoiding the partial glucose recovery observed in the C57BL/6 strain [15]. The accuracy of the DON study was enhanced by our establishment and maintenance of a reliable diabetes model.

Db/db mice were found to have hyperglycemia due to pancreatic *β*‐cell derangement at 4–8 weeks (i.e., 1–2 months old) and showed peak blood glucose at 3–4 months [16]. Previous histopathological studies showed no statistically significant difference in retinal structure between db/db and db/m groups at 8 weeks (younger than 2 months), indicating that the 2 month of age starting point in this experiment may be a node for significant differences in retinal structure between diabetic and control mice. Thus, at 2 months of age, db/db mice already exhibited early hyperglycemia but retained relatively preserved retinal morphology, making this an appropriate baseline for longitudinal observation of early DON development.

The latest DON expert consensus issued by the Neuro‐ophthalmology Group of the Chinese Medical Association stated that occult DON presents have normal visual acuity and normal fundus examination, but there are structural changes such as peripapillary RNFL thinning on OCT and visual function changes such as decreased contrast sensitivity. This suggests that DON can be diagnosed early using advanced imaging prior to the onset of clinical optic nerve changes. To validate the establishing of early DON model in our study, histopathological and molecular analyses further confirmed the involvements of pyroptosis and inflammation‐related proteins—characterized by upregulation of GSDMD and NLRP3—supporting the presence of early inflammatory neurodegeneration in the diabetic optic nerve.

### 4.2 Retinal Structure Alternations and Neurodegeneration

Prior experiments showed that the retinal thickness in db/db mice younger than 3 months old was significantly thinner than that of the db/m mice [17]. Another study found increased RGC apoptosis and optic nerve degeneration in db/db mice compared with controls after 20 weeks (younger than 5 months) and a marked thinning of TRT after 28 weeks (younger than 7 months) [18]. In addition, an FFA result from 28 weeks old db/db mice showed no significant DR manifestations such as vascular leakage, microaneurysms, or neovascularization in db/db mice [19]. While our results observed significant peripapillary RNFL thinning at 3 months old, and TRT thinning from initial state of hyperglycemia (2 months old) in db/db mice versus controls, which aligns with the hypothesis that neurodegeneration occurs early in diabetic retina [20].

The mechanism underlying retinal thinning in DON largely attributed to diabetes induced neuronal apoptosis. This process initially manifests in the RGCs within the ganglion cell layer, and amacrine cells located in the inner nuclear layer (INL) and inner plexiform layer (IPL) of the retina. As diabetes progresses, apoptotic activity increases in the photoreceptor cells (ellipsoid zone) of the outer retinal. These cumulative degenerative changes result in thinning of both the inner and outer retinal layers [21], affecting both RNFL and TRT as reported in our study. Such thinning strongly implies the presence of optic neurodegeneration. Furthermore, microvascular insufficiency may exacerbate peripapillary retina thinner and neurodegeneration even before overt DR lesions [22].

DON causes varying degrees of retinal thinning, which may also be related to different targeted areas. One study took around 4 mm diameter circle centered on the mouse ONH as ROI, which demonstrated that male 28‐week‐old db/db mice had significant thinning inner retinal layer compared with their control C57BL/KsJ db +/+ mice, but there was no significant difference in TRT [19]. However, RNFL‐INL thickness within a smaller sampling range in db/db mice aged 9 to 25 weeks was significantly thinner than in db/+ mice, and TRT was also significantly reduced, consistent with our findings [23]. Another study with a larger sampling area (6 mm diameter centered on the ONH) found that both OCT and H&E stained retinal sections showed that TRT was significantly decreased in diabetic mice (db/db) in comparison with nondiabetic (db/+) mice at 28 weeks. After 20 weeks of age, retinal staining indicated RGC cell apoptosis through TUNEL staining, but no OCT results were reported in the RNFL layer [18]. Therefore, a small sampling radius (0.234–0.324 mm) closer the ONH allowed us to detect subtle changes localized near the optic nerve more sensitively than broader regions used in previous studies.

Furthermore, previous studies had bred homozygous leptin knockout mice from the C57BKS strain with heterozygous transgenic mice from the C57BKS. To minimize genetic and phenotypic bias, we used phenotypically normal mice for breeding to avoid interstrain variation that can arise from crossing transgenic and non‐transgenic (C57BL6 vs. C57BKS).

### 4.3 Visual Function Decline and Structure—Function Correlations

Parallel to structural deficits, optic neurodegeneration also presents with abnormalities in visual behavior. Following RGC degeneration, both the number and viability of RGCs decline markedly. Secondary alterations occur in the interconnected bipolar, amacrine, and photoreceptor cells, which are adversely affected by ischemia and reduced metabolic support. These changes contribute to retinal thinning across multiple layers. Moreover, progressive retinal neurodegeneration disrupts optic nerve signal transmission, ultimately leading to functional visual deficits manifested as reduced visual acuity and impaired contrast sensitivity. A cross‐sectional study of Type 2 diabetes patients with DON without DR found visual impairment, defined as decreased visual acuity, reduced contrast sensitivity, and impaired color vision. Other visual function tests suggested that ERG results showed a lower b‐wave response in db/db mice compared to db/+ mice, indicating a diminished function of the rod photoreceptor and its associated neural pathways [23]. Here we found that db/db mice developed early DON and manifested progressive decline in visual acuity and increased contrast thresholds, whereas controls remained stable. It concluded that neuroretinal dysfunction was significantly associated with the presence of DON.

In this study, visual acuity was found to be significantly correlated with peripapillary TRT, suggesting that diabetes‐induced optic neuronal apoptosis may lead to functional impairments in visual acuity through the structural changes of peripapillary TRT. There were only few studies that have quantitatively analyzed the association between peripapillary TRT and BCVA [24], especially in the diabetic population. However, we found no correlation between contrast sensitivity and peripapillary TRT. Prior research has primarily reported a positive correlation between contrast sensitivity and macular ganglion cell complex thickness in patients with retinal neurodegeneration secondary to Type 2 diabetes [25]. A recent structure–function work suggests that peripapillary microvascular metrics (e.g., radial peripapillary capillary perfusion density) correlate with contrast sensitivity more robustly than thickness measures, which may explain our lack of association between contrast sensitivity and peripapillary TRT [26].

### 4.4 IOP And Systemic Influences

Early DON mice showed a significant increase in IOP from baseline at 2 months old and a decrease at 8 months old. Previous studies in db/db mice have generally observed IOP changes at 28 weeks old (younger than 7 months), suggesting that our findings capture the early phase of DON‐related IOP changes. Yet, evidence remains limited regarding whether early diabetic neurodegeneration itself leads to IOP elevation. Insulin resistance, a hallmark of Type 2 diabetes, has been positively correlated with increased IOP [27]. A study combined cross‐sectional and longitudinal changes have shown that body mass index (BMI) and changes in body weight were independently associated with IOP increases in obese patients after controlling for age, sex, and blood pressure [28]. These findings suggest that systemic metabolic factors—rather than local neurodegenerative processes—may play a dominant role in IOP regulation during early DON. Further studies are warranted to clarify how neuroinflammation and insulin resistance jointly modulate IOP in the early diabetic state.

### 4.5 Innovations, Shortcomings and Prospects

Unlike other cross‐sectional studies, this study has several methodological strengths, including its longitudinal design, precise ROI selection near the ONH, use of genetically matched controls (sharing the same ancestry as the experimental group), and integration of structural, functional, and histopathological assessments. This allowed us to detect the occurrence of early lesions quicker and to better clarify differences in the risks of adverse health outcomes between the experimental groups. These advantages enabled earlier detection of DON lesions and allowed for a clearer distinction in the risks of adverse neural outcomes between the experimental groups. The detection of RGC pyroptosis and inflammasome activation underscores inflammation‐mediated neural injury in the early pathogenesis of DON.

However, this study was limited to a 6‐month observation period. The RNFL and pathological changes began to show significant differences from the 3–4 months old, indicating that this study captured the early‐stage DON. Extending both earlier and later time points may uncover additional disease stages and potential reversibility in retinal structure and visual function within the course of DON. In future work, follow‐up beyond 8 months of age will be conducted to strengthen the temporal continuity and robustness of our findings. A study examining the retinal structure, function, and psychophysics in early diabetes emphasized that developing a reliable method to measure optic nerve injury has critical clinical implications for early prevention of irreversible stages of DON [29].

## 5 Conclusions

DON is a common blinding eye disease, which remains challenging to diagnose and manage due to limited understanding of its early pathophysiological mechanisms. Its later deterioration process of DON results in chronic and irreversible optic nerve impairments in clinical practice. Here, we initiated a controlled study using a noninvasive, multimodal evaluation system that combined OCT, optokinetic behavioral testing and IOP monitoring, validated by histopathological analysis. Our model assessed the extent of damages caused by early diabetic optic neurodegeneration in the peripapillary retina and their corresponding effects on visual function. The detection of inflammation‐mediated neuronal injury and RGC pyroptosis further elucidates key mechanisms underlying early DON. Overall, the successful establishment of this early DON evaluation model provides a valuable framework for investigating the onset and progression of diabetic optic neurodegeneration, offering significant potential to enhance early diagnosis and intervention, and to ultimately mitigate the detrimental effects of diabetes‐related optic nerve injury.

## Funding

This study was supported by the Beijing Administration Science & Technology Development of Traditional Chinese Medicine (Beijing, China): BJZYQN‐2023‐25; Scientific Incubating Foundation of Capital Medical University (Beijing, China): PYZ24115; the Priming Scientific Research Foundation for the Junior Researcher in Beijing Tongren Hospital, Capital Medical University (Beijing, China): 2021‐YJJ‐ZZL‐030.

## Conflicts of Interest

The authors declare no conflicts of interest.
